# TRAPPing Rab18 in lipid droplets

**DOI:** 10.15252/embj.201696287

**Published:** 2017-01-27

**Authors:** Francesca Zappa, Rossella Venditti, Maria Antonietta De Matteis

**Affiliations:** ^1^ Telethon Institute of Genetics and Medicine Pozzuoli Italy; ^2^ Department of Molecular Medicine and Medical Biotechnology University of Naples Federico II Naples Italy

**Keywords:** Membrane & Intracellular Transport

## Abstract

A number of membrane trafficking components are associated with lipid droplets (LDs) and/or are involved in their biogenesis. In this issue of *The EMBO Journal*, Li *et al* (2017) show that the mammalian TRAPPII (TRAnsport Protein Particle) complex acts as an LD‐associated GEF for Rab18, thereby regulating LD homeostasis.

Far from being simple storage structures, lipid droplets (LDs) are highly dynamic organelles that are involved in several functions. Testifying to their dynamic nature, several membrane trafficking components are linked to LDs. These include ARF guanine nucleotide exchange factors (GEFs), COPI components, and several Rab GTPases. Despite the fact that almost 40 different Rab GTPases have been found to associate with LDs, functional data regarding their role in LD biogenesis are available for only a few of them (Rab1, Rab5, Rab7, Rab8a, Rab32, Rab40c, and Rab18).

Rab18 associates with LDs, and it has been proposed to play a role in establishing the connection/exchange between the endoplasmic reticulum (ER) and the LD (Ozeki *et al*, 2005). The most convincing evidence supporting a role for Rab18 in LD homeostasis comes from the observation of an accumulation of enlarged lipid droplets in fibroblasts from patients affected by Warburg Micro syndrome, a neurological syndrome caused by mutations in Rab18, in Rab3GAP1/Rab3GAP2, which works as a GEF for Rab18, or in TBC1D20, a Rab18 GTPase‐activating protein (GAP) (Handley *et al*, 2015).

Rab18 also has a role in the maintenance of the ER and the Golgi complex (Gerondopoulos *et al*, 2014), and the activity of Rab3GAP1/Rab3GAP2 is required for the association of Rab18 with the endoplasmic reticulum (ER) (Handley *et al*, 2015). Therefore, the question remains whether the role of Rab18 in LD homeostasis is mediated by its function at the ER, or whether the activation of Rab18 can be regulated locally at the level of the LD by an LD‐associated Rab18 GEF.

In this issue of *The EMBO Journal*, Li and co‐workers provide an answer to this question. By adopting a combination of siRNA and gene‐editing experiments, they demonstrate that the mammalian TRAPP (TRAnsport Protein Particle) complex, and in particular TRAPPII, acts as a LD‐associated GEF for Rab18.

TRAPP, first identified in yeast almost 20 years ago, is a multisubunit modular complex consisting of core and peripheral subunits that is highly conserved from yeast to mammals. Several TRAPP complexes have been isolated in yeast (TRAPPI, TRAPPII, TRAPPIII, and, very recently, TRAPPIV) (Kim *et al*, 2016; Lipatova *et al*, 2016) sharing the same core subunits but containing distinct peripheral subunits. Only two TRAPP complexes, TRAPPII and TRAPPIII, have been described to date in mammals (Scrivens *et al*, 2011; Bassik *et al*, 2013).

The different TRAPP complexes in yeast take part in distinct trafficking events ranging from ER‐to‐Golgi transport (TRAPPI), late transport steps in the Golgi (TRAPPII), and autophagy (TRAPPIII and TRAPPIV). The picture is far less defined in mammals where TRAPPII has been found to play a role in intra‐Golgi and/or Golgi‐to‐PM transport (Yamasaki *et al*, 2009) and in ciliogenesis, while TRAPPIII has been shown to control ER‐to‐Golgi transport (Scrivens *et al*, 2011), autophagy (Imai *et al*, 2016; Lamb *et al*, 2016), human papilloma virus infection, and ricin toxicity (Bassik *et al*, 2013; Kim *et al*, 2016). In addition, a role in the ER export of fibrillar procollagen has been shown for TRAPPC2, a component that is shared by TRAPPII and TRAPPIII and the product of the gene mutated in the spondyloepiphyseal dysplasia tarda (Venditti *et al*, 2012) (Fig 1).

**Figure 1:**
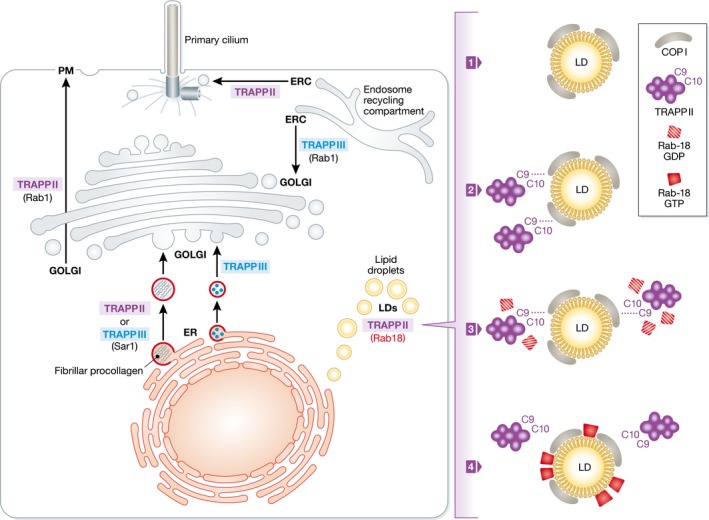
Sites of action of TRAPPII and TRAPPIII complexes in mammalian cells Two TRAPP complexes have been identified in mammalian cells, TRAPPII and TRAPPIII, which share common core subunits (TRAPPC1, TRAPPC2, TRAPPC3, TRAPPC4, TRAPPC5) but differ in the peripheral subunits: TRAPPII contains TRAPPC9 and TRAPPC10, while TRAPPIII contains TRAPPC8, TRAPPC11, TRAPPC12, and TRAPPC13. The scheme illustrates the different steps along the anterograde and retrograde membrane trafficking pathways that are under control of each TRAPP complex. In anterograde trafficking, TRAPPC2 is required for the ER export of fibrillar procollagens indicating a cargo‐specific role for TRAPPII and/or TRAPPIII in this step, TRAPPIII is required for ER‐to‐Golgi trafficking, while TRAPPII controls intra‐Golgi and/or Golgi‐to‐plasma membrane trafficking and the trafficking of ciliary proteins from endosomes to the primary cilium. In the retrograde pathway, TRAPPIII controls the trafficking from endosomes to the Golgi complex of different cargoes including ATG9, a key component of the autophagic machinery. The report by Li *et al* (2017) highlights a novel site of action for TRAPPII at lipid droplets. Also indicated are the Rabs activated by TRAPPII and by TRAPPIII at their different sites of action.

Initially proposed to act as a “passive” tethering factor, it was later shown that TRAPP complexes possess active “catalytic‐like” roles by acting as GEFs for GTPases of the Ypt/Rab family in both yeast and mammals (Kim *et al*, 2016).

In yeast, TRAPPI, TRAPPIII, and TRAPPIV can act as GEFs for Ypt1p (the homologue of Rab1) but sustain the role of Ypt1p in distinct processes, that is, ER‐to‐Golgi trafficking (TRAPPI) and autophagy (TRAPPIII, TRAPPIV), thus indicating the existence of pathway‐specific GEFs activating the same GTPase (Kim *et al*, 2016). Thus, the emerging scenario is that it is the GEF function of the TRAPPIII/IV complex that confers specificity on the activation of the same GTPase in different processes. The yeast TRAPPII complex instead acts as a GEF for Ypt31/32 (the homologues of Rab8/11). In mammals, both TRAPPII and TRAPPIII have been shown to act as a GEF for Rab1. With their findings, Li *et al* (2017) now demonstrate that the same TRAPPII complex acts as a GEF for another Rab GTPase, Rab18, at the level of LDs. The authors immune‐isolated TRAPPII and TRAPPIII using complex‐specific subunits (TRAPPC9 and TRAPPC12, respectively) and tested the GEF activity on recombinant Rab proteins *in vitro*. They found that the TRAPPII exchange activity on Rab1 and Rab18 is comparable, while TRAPPIII failed to activate either of them. The functional relevance of the GEF activity of TRAPPII on Rab18 is highlighted by the very similar LD phenotype (i.e., enlarged LDs) caused by either Rab18 or by TRAPPC9 depletion.

The site of action of TRAPPII as a GEF for Rab18 is at the LD where TRAPPII is recruited upon lipid load via its interaction with COPI (in particular through the interaction of the TRAPPII‐specific subunit TRAPPC9 with γ‐COP). These data therefore also highlight a new role for the LD‐associated COPI complex in addition to its proposed role in mediating the establishment of tubular connections between the ER and the LD (Wilfling *et al*, 2014).

While identifying a new molecular pathway fundamental for LD biogenesis, the work by Li *et al* (2017) also poses new questions: what is the relationship between the two Rab18 GEFs, that is, Rab3GAP1/Rab3GAP2 and TRAPPII? Do they intervene at different stages of LD biogenesis? Is LD‐associated TRAPPII responsible for the activation of other LD‐associated Rabs?

Finally, the data also provide the first example of a site of action of a mammalian TRAPP complex that is distinct from the endomembrane system, that is, in a lipid monolayer. It will be interesting to test in the future whether TRAPP is also found to participate in the biogenesis/assembly of other cellular structures not necessarily bound by a lipid bilayer.
